# AMPK: Potential Therapeutic Target for Vascular Calcification

**DOI:** 10.3389/fcvm.2021.670222

**Published:** 2021-05-11

**Authors:** Yi Lu, Tan Yuan, Xinjia Min, Zhen Yuan, Zhejun Cai

**Affiliations:** ^1^Department of Cardiology, The Second Affiliated Hospital, Zhejiang University School of Medicine, Hangzhou, China; ^2^Jiaxing Key Laboratory of Cardiac Rehabilitation, Jiaxing, China

**Keywords:** AMP-activated protein kinase, autophagy, endoplasmic reticulum stress, runt-related transcription factor 2, vascular calcification

## Abstract

Vascular calcification (VC) is an urgent worldwide health issue with no available medical treatment. It is an active cell-driven process by osteogenic differentiation of vascular cells with complex mechanisms. The AMP-activated protein kinase (AMPK) serves as the master sensor of cellular energy status. Accumulating evidence reveals the vital role of AMPK in VC progression. AMPK is involved in VC in various ways, including inhibiting runt-related transcription factor 2 signaling pathways, triggering autophagy, attenuating endoplasmic reticulum stress and dynamic-related protein 1-mediated mitochondrial fission, and activating endothelial nitric oxide synthase. AMPK activators, like metformin, are associated with reduced calcification deposits in certain groups of patients, indicating that AMPK is a potential therapeutic target for VC.

## Introduction

Vascular calcification (VC) is characterized by accumulating calcium deposits in the tunica intima and tunica media of the vessel wall. The mineral deposition results in stiffness of conduit arteries and impaired elasticity of vessels that preserve distal perfusion during the cardiac cycle (1). The burden of VC, especially coronary artery calcification, is an independent risk factor for cardiovascular events and long-term all-cause mortality (2), which constitutes a critical medical problem with aggregating economic burden (3). However, due to the complexity of the underlying mechanism of VC (4), invasive transcatheter procedures and surgeries are the only available options for severe calcific vascular diseases (5), and there is no clinically approved medical therapy for VC, so far.

VC was once deemed as a passive, unregulated, degenerative process in the past (6). However, accumulating evidence suggests that VC is an active cell-driven process (6, 7). Vascular smooth muscle cells (VSMCs) are the essential constituents of the vascular wall. Those contraction-related proteins secreted by contractile type of VSMCs are critical for regulating blood pressure and maintaining the extracellular matrix (ECM) of vessels (8). However, the contractile phenotype is predisposed to osteoblastic phenotypic transition under certain local stimuli like inflammation (9). This phenotypic transdifferentiation is a hallmark in the pathogenesis of VC, which is characterized by the loss of contraction-related proteins and the accumulation of osteoblastic-involved proteins, including runt-related transcription factor 2 (Runx2), alkaline phosphatase (ALP), and osteopontin (9–11).

The AMP-activated protein kinase (AMPK) is an evolutionarily conserved serine/threonine-protein kinase. It is a heterotrimeric complex consisting of a catalytic α subunit and two regulatory β and γ subunits (12). The α subunit has two isoforms (α1 and α2), which are differentially distributed in different cells (13). AMPK serves as a critical cellular energy sensor that is expressed ubiquitously in eukaryotic cells (14). Knowledge of AMPK upstream inducers and downstream targets is expanding (15, 16). AMPK can be activated via canonical and non-canonical mechanisms (16). The increase of AMP: ATP and ADP: ATP ratio during cellular energy stress triggers AMPK through canonical mechanisms (16). 5-aminoimidazole-4-carboxamide ribonucleoside (AICAR), which can be converted into AMP analogs that bind the γ subunit of AMPK, is wildly used in laboratory research as a non-canonical AMPK activator. Intracellular Ca^2+^ can activate AMPK by phosphorylating Thr172 by calmodulin-dependent protein kinase CaMKKβ (17, 18). Both canonical and non-canonical mechanisms are involved in the Ca^2+^/CaMKKβ pathway. What is more, multiple commercialized drugs, like metformin, simvastatin, and resveratrol, can activate AMPK indirectly by inhibiting ATP synthesis (15).

Besides the classical role of AMPK in metabolic regulation, increasing evidence indicates that AMPK is a crucial player in the pathogenesis of VC. Pharmacological activation of AMPK can significantly inhibit VSMC calcification (19, 20). Treatment of AMPK activator metformin was associated with lower coronary and extremity artery calcification burden in diabetic patients (21, 22). In this review, we will focus on the recent advances concerning the role of AMPK in VC and interpret its potential therapeutic utility.

## The Protective Role of AMPK Against VC

### AMPK-Runx2 Signaling Pathways

Runx2, also named Core-binding factor alpha 3 subunit (CBFA1), is a well-accepted activator of osteoblast differentiation-related genes (23). The elevated expression of Runx2, together with other osteoclastic-associated proteins, in calcified human vascular tissues (11) and mice VC models (24, 25), cast light on its role in vascular calcification.

The relationship between AMPK and Runx2 was first reported in osteogenesis (26, 27). Jang et al. found that metformin increased the expression of Runx2 via AMPK, which resulted in the stimulation of osteoblast differentiation (27). However, AMPK-Runx2 signaling pathway seemingly exerts an opposite role in VSMCs differentiation. Previous studies had demonstrated that Runx2 was expressed in VSMCs rather than macrophages in the calcified lesions (19, 28), and VSMC autonomous Runx2 was essential for vascular osteogenesis (28). Vascular calcification was markedly inhibited in VSMC-specific Runx2-deficiency mice fed with high-fat diet, which was accompanied by decreased macrophage infiltration and osteogenic differentiation (29). Cao et al. investigated AMPK-Runx2 in VSMCs and reported that AMPK activation downregulated the Runx2 expression in VSMCs (30). Our previous work further investigated the underlying mechanisms. We found that the activation of AMPKα1 could phosphorylate PIAS1, the SUMO E3-ligase of Runx2, to enhance the instability of Runx2. Moreover, deficiency of AMPKα1 in VSMC resulted in the upregulated expression of Runx2 and promoted osteoblastic differentiation of VSMCs. On the other hand, chronic metformin treatment could prevent the VC process and down-regulate Runx2 level in *Apoe*^−/−^ mice through activating AMPKα1 (19). One possible explanation for the opposite roles of AMPK-Runx2 in osteoblasts and VSMC differentiation might be the different responses of Smurf1 (the ubiquitin E3-ligase of Runx2) upon AMPK phosphorylation (19, 31).

Besides the direct effect of AMPK-Runx2 pathway on VSMC transdifferentiation, a recent study indicated that AMPK could also inhibit VC by regulating receptor activator of nuclear factor kappa-B ligand (RANKL) (32). RANKL serves as a chemoattractant that induces the infiltration of macrophage and the transformation of macrophage into bone-resorbing osteoclast-like cells, which further accelerate the process of VC (29, 32, 33). Since RANKL is a known downstream factor of Runx2 (34), it is reasonable to hypothesize that Runx2 mediates the AMPK-RANKL pathway.

To sum up, the activation of AMPK could down-regulate the expression and activity of Runx2 both in the translational and post-translational levels, which results in the inhibition of osteoblastic differentiation of VSMCs. Besides, AMPK-Runx2 signaling pathway may be involved in the infiltration and transformation of macrophages by down-regulating the level of RANKL.

### AMPK and Autophagy Pathways

Autophagy is indispensable for human health by degrading cellular components like dysfunctional proteins or organelles in lysosomes. This catabolic process is up-regulated under specific stimuli like nutrient deprivation (35), resulting in cellular death and metabolic stress (15). Recent evidence suggests that autophagy was also implicated in VC development (36–38). Autophagy is enhanced in VC models, and the activation of autophagy ameliorates the pathology of VC both in calcified VSMCs (39) and rat VC models (40). One possible explanation is autophagy could inhibit the apoptosis and osteoblastic transformation of VSMCs (41).

AMPK is an integral part of autophagy with complex mechanisms. By phosphorylating tuberous sclerosis complex 2 (42), subunit raptor of the mechanistic target of rapamycin (mTOR) (43) and Unc-51-like kinase 1 (ULK1) (44), AMPK initiates autophagy directly (15). Meanwhile, by regulating the expression of relevant downstream transcription factors like FOXO3, AMPK can also initiate autophagy indirectly (45). Recent studies had investigated the protective role of autophagy in VC through AMPK activation. By activating AMPK with melatonin (41) or ghrelin (36), the process of autophagy was enhanced, which resulted in reduced VSMC osteoblastic differentiation both in cell culture (41) and rat models of VC (36). On the other hand, treatment of aldosterone or advanced glycation end products (AGEs) facilitated VC by inhibiting AMPK-dependent autophagy (46). Pretreatment of AMPK activator AICAR could upregulate the autophagy level and reverse the effect of AGEs on osteoblastic differentiation of VSMCs (41, 47). AMPK/mTOR signaling pathway was the possible involving mechanism (41, 47, 48).

Taken together, AMPK activation was associated with the enhancement of autophagy and, subsequently, inhibited VSMC calcification. However, most of the studies mentioned above were conducted in cultured VSMCs. More studies with direct evidence are needed to verify these findings *in vivo*.

### AMPK and Endoplasmic Reticulum Stress

The endoplasmic reticulum (ER) is an essential intracellular organelle that acts as a protein synthesis factory. ER has pivotal roles in coordinating energetic disturbance via regulating metabolism and cell fate decisions (49). The disruption of ER homeostasis is defined as ER stress (ERS), resulting in the activation of PKR-like ER kinase (PERK), inositol-requiring enzyme 1 (IRE1), and activating transcription factor 6 (ATF6), which causes an adaptive signaling pathway named unfolded protein response (UPR) (50, 51). Prolonged ER stress ultimately leads to the modulation of multiple cellular pathways, including apoptosis, necroptosis, autophagy, and UPR-associated morphological changes (49).

Increasing evidence reveals the tight connection between AMPK activation and ERS in different disease models (52–54). AMPK antagonists abolished deficiency-mediated inhibition of ERS in VSMCs incubated with calcifying media (55). Biomarkers of ERS were increased significantly in calcification lesions (56, 57) and were associated with VSMC apoptosis (56). Compared with *Apoe*^−/−^ littermates, the ERS and prevalence of atherosclerosis was significantly increased in *Apoe*^−/−^*Prkaa2*^−/−^ mice (58, 59). However, as the last stage of atherosclerosis, the role of AMPK in ERS-mediated vascular calcification has not been fully elucidated.

Among all the effector molecules in response to ERS, transcription factor 4 (ATF4) is proved to be of enormous significance in VC. ATF4 was up-regulated in calcified aortas and VSMCs, while inhibition of ERS alleviated calcification (55). Previous studies found that PERK-eukaryotic initiation factor 2α(eIF2α)-ATF4 signaling pathway was involved in ERS-induced VSMCs apoptosis and osteoblast differentiation during the process of VC (60, 61). Li et al. found that death-associated protein kinase 3 (DAPK3) inactivated AMPK signaling and promote the expression of ERS-related protein (including ATF4), thus leading to osteogenic differentiation of VSMCs and VC (55).

In summary, the activation of AMPK inhibits ERS to ameliorate VC. ERS downstream protein like ATF4 mediates phenotypic transformation and apoptosis of VSMC that promotes the VC process. More specific inhibitors like ATF4 inhibitors may be further validated in clinical application in VC.

### AMPK-eNOS-NO Signaling Pathway

Endothelium-derived nitric oxide (NO) is a messenger molecule that is crucial in the maintenance of vascular function (62, 63). Endogenous NO functions as a modulator of VSMC proliferation and migration (64, 65), which can inhibit VC by interfering with transforming growth factor-beta (TGF-β) signaling (66). Endothelial nitric oxide synthase (eNOS) is the primary enzyme for NO production in endothelial cells. Genetic lack of eNOS was associated with raised atherosclerotic lesions and valvular calcification in mice models (67, 68). A recent study revealed that eNOS deficiency was also associated with the exacerbation of aortic calcification (69). On the other hand, exercise training prevented eNOS down-regulation and resulted in fewer calcification deposits in rat VC models (70). However, in contrast to previous findings that eNOS mainly play a protective role in VC, Tziakas et al. found that erythrocyte-origin eNOS might be harmful in the development of VC (71).

AMPK is a well-defined regulator of eNOS. By phosphorylating eNOS at Ser1177/1179, AMPK enhances the activity of eNOS in a post-translation manner (72). Kanazawa et al. found that metformin could induce the differentiation and mineralization of osteoblasts via activating AMPK (73). The elevated AMPK expression protects human coronary artery endothelial cells from diabetic lipoapoptosis via increasing eNOS synthesis (74). Daily injections of AMPK activator AICAR attenuated high-fat diet-induced arterial stiffening in Klotho-deficient mice, together with increased level of phosphorylated eNOS (75). Besides endothelial cells, VSMCs are also known origins of eNOS (66). Cao et al. explored the underlying mechanism in rat aortic VSMCs with a β-glycerophosphate-induced VC model and found that metformin-mediated calcification protection was AMPK-eNOS-NO-dependent (30). AMPK activation by metformin treatment was accompanied by increased eNOS level and NO overproduction (30, 76). Inhibition of either AMPK or eNOS abolished metformin-mediated VC prevention, indicating an essential protective role of the AMPK-eNOS-NO pathway in VC development (30). Due to the limitation of *in vitro* study, further study is needed to verify the protective role of AMPK-eNOS-NO in VC in animal models.

These findings suggest the activation of AMPK-eNOS-NO signaling pathway is associated with the amelioration of VC. Since endothelial cells rather than VSMCs are the primary source of eNOS, it is more reasonable that AMPK acts on endothelial cells' eNOS signaling to prevent VC.

### AMPK and Mitochondrial Dynamics

Mitochondria are continually undergoing fission and fusion, termed as mitochondrial dynamics, under the control of specific fission and fusion machinery (77, 78). A proper balance in mitochondrial dynamics is critical for mitochondrial morphology, biogenesis, degradation, and cellular apoptosis (79, 80). Recruitment of dynamic-related protein 1 (DRP1) from the cytosol is required in mitochondrial fission, which causes constriction and eventual division of the mitochondria (78, 81). Not until recently did scientists uncovered that DRP1 promoted cardiovascular calcification via regulating osteogenic differentiation (82). The inhibition of expression and phosphorylation of DRP1 ameliorated the apoptosis of VSMC and attenuated VC in rodent VC models (83, 84).

AMPK is genetically required for cells to process rapid mitochondrial fission. By direct phosphorylating mitochondrial fission factor (MFF, the dominant receptor of DRP1), AMPK can acutely trigger mitochondrial fission (85, 86). Also, Drp1 is a known downstream factor of AMPK. In addition to regulating the expression of Drp1, AMPK can phosphorylate DRP1 at Ser-637, resulting in the inhibition of Drp1 activity and its translocation to mitochondria (87, 88). Previous studies had shown that AMPK activation could suppress atherosclerosis and endothelial dysfunction by reducing DRP1-mediated mitochondrial fission (88, 89). Activation of AMPK by metformin reduced DRP1 expression, mitochondrial fragmentation, and plaque formation in diabetic mice models, while AMPKα2 deficiency abolished the effect s of metformin on atherosclerosis in *Apoe*^−/−^*Prkaa2*^−/−^ mice. Another recently published work described the protective role of the AMPK-DRP1 pathway in the calcification of VSMCs (90). By activating AMPK expression with melatonin, the expression of Drp1 was decreased and subsequently inhibited mitochondrial fission, which resulted in reduced apoptosis, Runx2 expression, and calcium deposition (90).

In general, AMPK-mediated mitochondrial fission attenuates VC by inhibiting the expression of DRP1. However, the role of mitochondrial dynamics in VC has not been fully elucidated. More studies are needed to prove the function of AMPK-dependent DRP1-mediated mitochondrial fission in VC. DRP1 activators may also be applied as a target for VC treatment.

## Endogenous AMPK Activator and VC

As mentioned above, the expression and activity of AMPK are under strict and delicate regulation, which is tightly associated with ATP metabolism. ATP is metabolized by ENPP1 into AMP and pyrophosphate, which was then further hydrolyzed by CD73 to build adenosine and phosphate (91). Phosphate, calcium, pyrophosphate (PPi), and adenosine are important inducers that activate AMPK canonically. These endogenous AMPK activators can also influence the process of VC. High phosphate level is considered the main determinants of VC in patients with chronic kidney disease (92). Hyperphosphatemic triggers diverse signaling pathways (including Runx2) that enhance the sensitivity of VSMCs to calcification (93), and reduces levels of calcification inhibitors (94). On the other hand, PPi is a well-accept endogenous inhibitor of biomineralization (95). The presence of PPi inhibits the calcification of rat aortas *in vitro* (96). Humans lacking ectonucleotide pyrophosphorylase1 (extracellular PPi synthesizer) develop severe VC at an early age (97). What is more, recent studies demonstrate that adenosine might serve as an endogenous inhibitor of VC through regulating the expression of tissue non-specific alkaline phosphatase (TNAP) (98, 99).

## Applications of AMPK in VC Clinical Settings

Accumulating evidence shows that VC is an active cell-driven process (6, 7), which poses a potential for therapeutic targeting (41). Considering the protective effects of AMPK in VC, it is reasonable to speculate that AMPK activators can prevent VC. Multiple communalized drugs can activate AMPK indirectly, either by inhibiting ATP synthesis (like metformin, statins) (16, 100), or by inhibiting tetrahydrofolate-utilizing enzymes that catalyze ZMP to purine nucleotides (like pemetrexed, methotrexate) (16, 101). Since pemetrexed and methotrexate function as immune suppressors, we mainly discuss drugs, including metformin and statins in current review.

Metformin, the first-line oral anti-diabetic drug, can activate AMPK in a dose- and time-dependent manner both *in vivo* and *in vitro* (73, 102, 103), which might be implicated in VC treatment and diabetic complications prevention (104). Metformin prescription resulted in a significant reduction of circulating osteoprotegerin, a biomarker of VC, in diabetic patients (105). The VC progression in the coronary artery and peripheral artery was inhibited by metformin usage (22, 106). Our group had reaffirmed that metformin prescription was associated with lower coronary artery calcification levels among patients with type 2 diabetic mellitus (T2DM) (21). The association was independent of age, gender, duration of T2DM and renal function (21). Another group investigated metformin usage in prediabetic patients, which demonstrated its consistent VC protective effect in male prediabetic subjects (107). We believe that metformin's protective effect on VC, which was seemingly independent of serum glucose, is at least partly achieved through AMPK activation (21).

## Discussion and Perspectives

**Figure 1:**
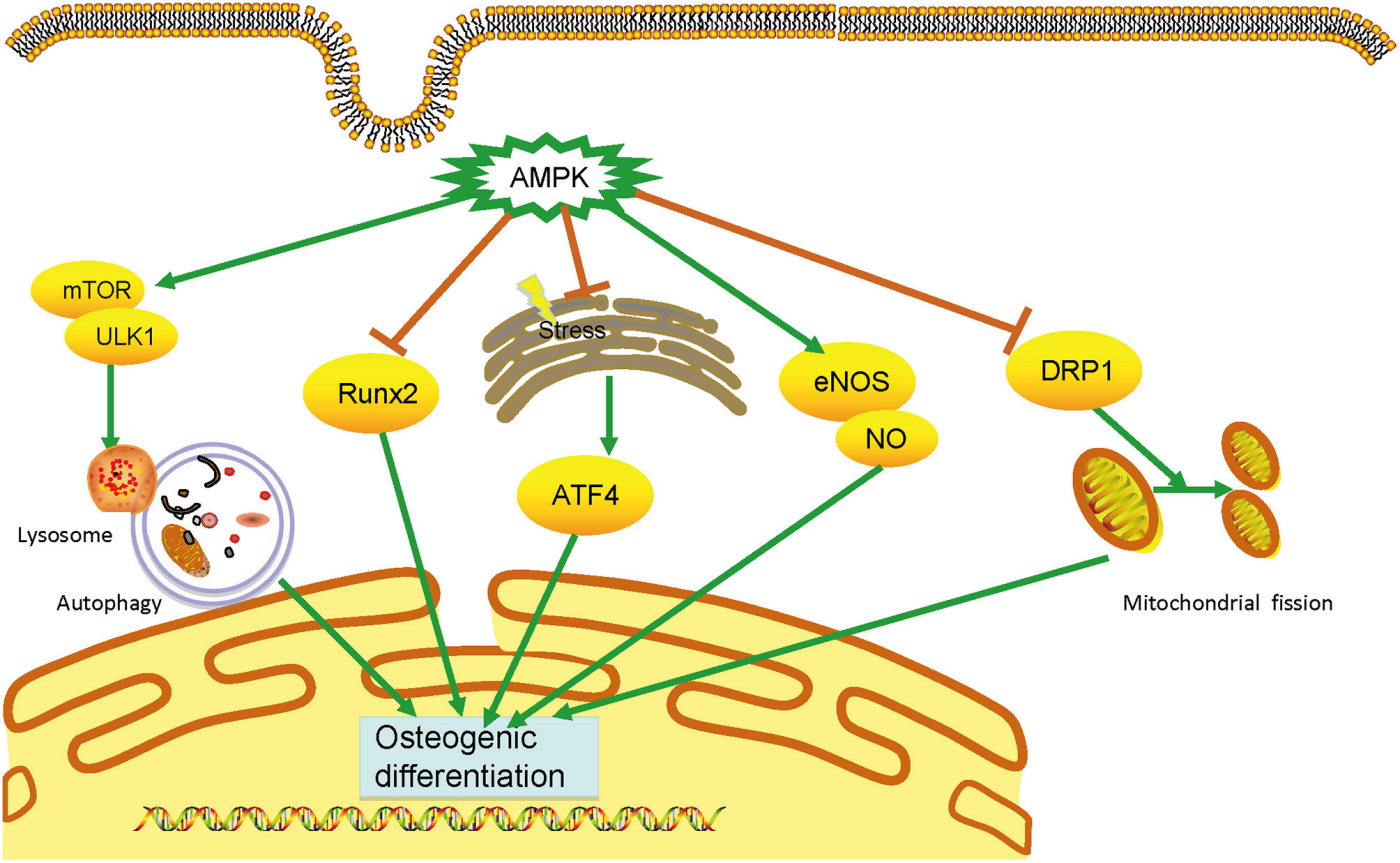
Mechanisms of AMPK in vascular calcification. AMPK plays an essential role in calcification through multiple mechanisms, including inhibiting runt-related transcription factor 2 (Runx2) signaling pathways, triggering autophagy, attenuating endoplasmic reticulum stress, and dynamic-related protein 1(DRP1)-mediated mitochondrial fission, and activating endothelial nitric oxide synthase. By preventing the osteogenic differentiation of vascular smooth muscle cells, AMPK can prevent vascular calcification development. AMPK, AMP-activated protein kinase; DRP1, dynamic-related protein 1; eNOS, endothelial nitric oxide synthase; ERS, endoplasmic reticulum stress; VSMC, vascular smooth muscle cell.

VC is an urgent worldwide health issue with no available medical treatment. Accumulating evidence shows that AMPK plays a vital and protective role in developing VC via distinct signaling pathways, including the Runx2, autophagy, ERS, eNOS activation, and DRP1 (Figure 1).

Activation of AMPK by medicines is a potential therapeutic approach for vascular calcification. However, there are still many unanswered questions in the field. Agents that precisely targeting AMPK or the subunits of AMPK are yet to be developed. While metformin is mainly prescribed for diabetes patients, it will be interesting to determine metformin's effect on VC in otherwise non-diabetic subjects. Whether AMPK activation could prevent or reverse the pathological process of VC needs to be explored. For conditions like chronic kidney disease prone to develop severe VC, whether AMPK activation is protective against VC is unknown. More clinical studies, especially prospective randomized clinical trials, are required to confirm the therapeutic target of AMPK for VC.

## Author Contributions

ZC and YL designed the study. YL and TY drafted the manuscript. XM and ZY contributed data. All authors were involved in critically revising the manuscript.

## Conflict of Interest

The authors declare that the research was conducted in the absence of any commercial or financial relationships that could be construed as a potential conflict of interest.
